# Dose Optimization and Targeting Strategies of Anti-Infective Agents

**DOI:** 10.3390/pharmaceutics15071870

**Published:** 2023-07-03

**Authors:** Martin Šíma, Ondřej Slanař

**Affiliations:** Department of Pharmacology, First Faculty of Medicine, Charles University and General University Hospital in Prague, 128 00 Prague, Czech Republic; ondrej.slanar@lf1.cuni.cz

Most drugs are currently developed, approved and marketed based on their effect on the majority of the population. However, the concept of a single dose of a drug for all patients with the same disease may not lead to the desired results. The concentration profile of a drug over time after the same dose administered to individual patients may vary significantly. The same dose may thus lead to signs of toxicity in some patients, while in others, it may be ineffective. In addition, for anti-infective agents, low exposure may be accompanied by the development of resistance. In order to achieve the maximum therapeutic potential of anti-infective drugs, the therapy needs to be targeted and the dosage optimized. This Special Issue, therefore, aims to serve as a platform for sharing the contemporary progress in dose optimization and targeting strategies.

The research section includes one review and four original research papers. The review from Huntjens et al. summarize the current evidence about pharmacokinetic and pharmacodynamics properties of antiviral agents for herpes simplex virus and cytomegalovirus treatment in immunocompromised patients [1]. Given the narrow therapeutic window of antivirals and the fragility of immunocompromised patients, it is not surprising that the authors advocate therapeutic drug monitoring as the standard in this therapy. In addition, there is a discussion on, e.g., the early recognition of drug resistance, the implementation of new antivirals, the alternation of agents with different toxicity profiles and the combination of synergistic antivirals. Katip et al. compared the clinical outcomes of patients receiving a loading dose of colistin-meropenem and a loading dose of colistin-imipenem in the treatment of carbapenem-resistant *Acinetobacter baumannii* infection [2]. This study showed that patients receiving colistin-imipenem had a lower 30-day survival rate and a lower clinical and microbiological response compared with patients treated with colistin-meropenem, while no significant difference in nephrotoxicity was observed between the two combined regimens. The pharmacokinetic study of Sima et al. described the exposure to the main active metabolites of ciprofloxacin in critically ill patients and identified several factors affecting ciprofloxacin/its active metabolite ratios [3]. Based on these data, a parent drug-metabolite population pharmacokinetic model for ciprofloxacin/desethylene ciprofloxacin has subsequently been developed. Since the metabolite elimination rate constant (and not the parent drug to metabolite rate constant) is increased in CYP1A2 rs762551 variant allele carriers, it can be hypothesized that CYP1A2 inhibition by ciprofloxacin is mediated by its metabolite, desethylene ciprofloxacin. It is a demonstration of a new approach, where a population pharmacokinetic model proposed a valid mechanistic target for a drug–drug interaction study. The paper of Zhang et al. used population pharmacokinetic simulations of clinical dosing regimens to predict the impact of aztreonam-amoxicillin-clavulanate combination on restricting mutant selection of New Delhi metallo-β-lactamase and serine-β-lactamase co-producing *Escherichia coli* and *Klebsiella pneumoniae* [4]. Based on simulations, this combination has limited coverage against New Delhi metallo-β-lactamase- and extended-spectrum β-lactamase co-producing *E. coli* and *K. pneumoniae* and is not effective against isolates carrying plasmid-mediated AmpC and KPC-2 β-lactamases. Peritoneal dialysis is a specific modality of renal replacement therapy. In their study, Hartinger et al. dealt with intraperitoneally administered vancomycin in peritoneal dialysis-associated peritonitis patients [5]. The first-ever population pharmacokinetic model for intraperitoneal vancomycin administration was developed and vancomycin exposure after dosing schedules recommended by the International Society for Peritoneal Dialysis was simulated. Simulations showed that recommended dosing schedules may lead to underexposure of a large proportion of patients. To prevent this, a new continuous dosing regimen was suggested consisting of a loading dose of 20 mg/kg followed by maintenance doses of 50 mg/L in each dwell.

Searching the Pubmed database, we have found that the annual number of articles for the keyword “dose optimization” increased more than fivefold between 2000 and 2022. From the formerly widely used so-called two-step approach, where individual pharmacokinetic parameters were first calculated and then associated with the covariates considered [6], we have progressively shifted to the use of a mainly population-based approach, where the concentration-time profiles of a population are analyzed as a whole. The final model provides typical population pharmacokinetic parameters and describes interindividual and intraindividual variability that may be explained by covariates [7]. For example, three of the four original articles in this Special Issue used a population analysis. The widespread implementation of these advanced statistical methods in the process of pharmacokinetic-based dose optimization has enabled further scope for tailored pharmacotherapy. We believe this trend will continue to expand and become an integral part of dosing recommendations across a wide variety of medical disciplines, ultimately bringing benefit to patients.
